# Zinc Blockade of SOS Response Inhibits Horizontal Transfer of Antibiotic Resistance Genes in Enteric Bacteria

**DOI:** 10.3389/fcimb.2018.00410

**Published:** 2018-11-21

**Authors:** John K. Crane, Muhammad B. Cheema, Michael A. Olyer, Mark D. Sutton

**Affiliations:** ^1^Division of Infectious Diseases, Department of Medicine, Jacobs School of Medicine and Biomedical Sciences, University at Buffalo, Buffalo, NY, United States; ^2^Department of Biochemistry, Jacobs School of Medicine and Biomedical Sciences, University at Buffalo, Buffalo, NY, United States

**Keywords:** antibiotic resistance, RecA, electrophoretic mobility shift assay, *Enterobacter cloacae*, extended spectrum beta lactamase, CTX-M27, Zinc pyrithione

## Abstract

The SOS response is a conserved response to DNA damage that is found in Gram-negative and Gram-positive bacteria. When DNA damage is sustained and severe, activation of error-prone DNA polymerases can induce a higher mutation rate than is normally observed, which is called the SOS mutator phenotype or hypermutation. We previously showed that zinc blocked the hypermutation response induced by quinolone antibiotics and mitomycin C in *Escherichia coli* and *Klebsiella pneumoniae*. In this study, we demonstrate that zinc blocks the SOS-induced development of chloramphenicol resistance in *Enterobacter cloacae*. Zinc also blocked the transfer of an extended spectrum beta-lactamase (ESBL) gene from *Enterobacter* to a susceptible *E. coli* strain. A zinc ionophore, zinc pyrithione, was ~100-fold more potent than zinc salts in inhibition of ciprofloxacin-induced hypermutation in *E. cloacae*. Other divalent metals, such as iron and manganese, failed to inhibit these responses. Electrophoretic mobility shift assays (EMSAs) revealed that zinc, but not iron or manganese, blocked the ability of the *E. coli* RecA protein to bind to single-stranded DNA, an important early step in the recognition of DNA damage in enteric bacteria. This suggests a mechanism for zinc's inhibitory effects on bacterial SOS responses, including hypermutation.

## Introduction

The term “SOS response” as descriptor of a bacterial reactions to DNA damage was coined about 1975, the same year that the ship Edmund Fitzgerald sank in Lake Superior without sending out an SOS distress call. The SOS response triggers a constellation of changes in the bacterial cell, including cellular elongation, suspension of cell division, induction of DNA repair pathways, induction of latent bacteriophages, expression of bacteriophage-encoded toxins, and increased expression of error-prone DNA polymerases (Goodman, 2002). The latter underlies the increased mutation rate observed when the SOS response is strongly induced.

Recently, several laboratories showed that sublethal concentrations of certain SOS-inducing antibiotics induced resistance to other, unrelated antibiotics (Kohanski et al., 2010; Song et al., 2016; Bunnell et al., 2017). Their work and that of others have led to increased interest in whether SOS inhibitors could block emergence of antibiotic resistance (Nautiyal et al., 2014; Alam et al., 2016).

We initially became interested in the SOS response because of its role in inducing the expression of the Shiga toxins in Shiga-toxigenic *Escherichia coli* (STEC), because antibiotics induce production of the Shiga toxins Stx1 and Stx2, resulting in a worsening of the disease. Since previous work showed that zinc inhibited Stx production in STEC, we wondered if zinc's inhibitory effects were mediated by inhibition of the SOS response, and found that indeed they were (Crane et al., 2014). Indeed, zinc's inhibitory effects on the SOS response also blocked hypermutation in *E. coli* and *Klebsiella* strains (Bunnell et al., 2017).

Beaber et al. previously showed that induction of the SOS response with mitomycin C or ciprofloxacin dramatically increased the rate of horizontal antibiotic resistance transfer from *E. coli* to *Vibrio cholerae*, and from one strain of *V. cholerae* to another (Beaber et al., 2003). In this study, we first tested whether the inhibitory effects of zinc salts on SOS also extended to another member of the Enterobacteriaceae family, *Enterobacter cloacae*. We next tested whether zinc ionophores, such as A23187 or zinc pyrithione, would show increased potency against the hypermutation response. We also tested whether zinc could block the transfer of antibiotic resistance from *Enterobacter* to a susceptible *E. coli* recipient strain. Zinc salts and zinc pyrithione blocked hypermutation in *E. cloacae* and zinc also blocked horizontal transfer of ß-lactam resistance to another, unrelated *E. coli* strain. Last, we developed an electrophoretic mobility shift assay (EMSA) to determine if metals blocked binding of RecA to ssDNA. We demonstrated that zinc, but not other divalent metals, blocked the ability of RecA to bind to ssDNA in this EMSA.

## Materials and methods

### Materials

Mitomycin C, ciprofloxacin (cipro), chloramphenicol (chloramph), zinc acetate, zinc pyrithione, A23187, agarose, ATP, ATP-γ-S, Sypro Orange protein stain, and copper phthalocyanine tetrasulfonate were from Sigma-Aldrich (St. Louis, MO). Gradient-impregnated antibiotic test strips were purchased from Liofilchem, Inc., (Abruzzi, Italy, and Waltham. MA). Copper pyrithione was from Combi-Blocks, San Diego, CA, while ceftazidime (ceftaz) was purchased from TCI, Tokyo, Japan. DMEM-F12 medium was from Gibco, Inc., Grand Island, NY, a subsidiary of Thermo-Fisher. DNase I was from Worthington Biochemical Corp., Lakewood, NJ. Purified RecA protein was purchased from New England Biolabs (NEB, Boston, MA). Blue Juice loading dye, SYBR-Safe DNA dye, and the 100 bp DNA ladder were from the Invitrogen division of Thermo-Fisher.

Bacterial Strains Used. Bacterial strains are described in Table 1.

**Table 1:** Description of bacterial strains used.

	**Description**	**Ciprofloxacin MIC on DMEM, mg/L**	**Chloramphenicol MIC on LB agar, mg/L**	**Ceftazidime MIC on LB agar, mg/L**	**Comments**
**ENTEROBACTER STRAINS**					
*Enterobacter cloacae* E_clo_Niagara	Wild-type, clinical isolate, bloodstream infection, ESBL	0.064	4	6 on LB; ≥ 16 on Muller-Hinton	“ESBL” or extended spectrum beta- lactamase producer; + ^for^ *bla*_CTX−M27_
BAA-1143	ATCC type strain	0.006	8	96	AmpC chromosomal beta-lactamase; from Microbiologics, St. Cloud, MN
***E. coli*** **STRAINS**					
*E. coli* EC43	O157:H7 (pGFP-UV-Chloramph^R^). Expresses green fluorescent protein (GFP) from plasmid; Chloramphenicol–resistant.	0.008	>40	0.064	Microbiologics, St. Cloud, MN; traceable to FDA strain ESC1177

### Hypermutation in *E. cloacae* bacteria

As previously described, bacteria were grown overnight in LB broth at 37°C with 300 rpm shaking, then subcultured into DMEM-F12 broth medium for induction of hypermutation. The DMEM/F12 medium was supplemented with 18 mM NaHCO_3_ and 25 mM additional HEPES, pH 7.4, but omitted serum and antibiotics. The usual dilution from overnight was 1:100 into DMEM-F12, but this was varied if needed. After 75 min, the culture was divided into separate tubes and ciprofloxacin was added to a concentration equivalent to ½ to ¾ of the MIC for that strain. Differences in the concentration of ciprofloxacin used resulted in differences in the antibiotic resistance frequencies observed. Metals such as zinc were added at this time, and incubation was continued with 300 rpm shaking. Four hours after initiation of the subculture, the culture turbidities were measured as OD_600_ on a spectrophotometer, and serial dilutions were quickly plated onto LB agar to measure the total bacterial counts in each condition. In addition, 100–200 μl of undiluted culture was plated in triplicate onto plates of LB + 20 mg/L (20 μg/mL) chloramphenicol, a concentration 5 times the MIC of E_clo_Niagara. Antibiotic resistance frequencies were calculated as the (CFU of antibiotic resistant colonies/mL) ÷ (CFU of total colonies/mL). In some cases, the antibiotic resistance frequency was multiplied by 10e6 and expressed as “chloramphenicol resistance frequency per million” as previously shown, and in other cases these results were converted to a logarithmic scale. When zero colonies were observed on any of the triplicate plates, the lower limit of detection was calculated as if 1 colony had been observed; this was often the case in the control cultures, i.e., bacteria not exposed to any SOS-inducing antibiotic.

### Cross-species (horizontal) transfer of antibiotic resistance

In order to test for the transfer of genetic material from the ceftaz^R^ *E. cloacae* to Chloramph^R^ fluorescent *E. coli* strain EC43, or vice versa, both strains were grown up in LB broth overnight, then both were subcultured into DMEM-F12 liquid medium. After 75 min, ciprofloxacin was added at the appropriate concentrations for each strain. At this time, metals such as zinc were also added. At the 4 h time point, the culture turbidities were read as OD_600_, and then dilutions were made in order to determine the total number of bacteria of each species in their separate cultures. Bacteria were then mixed in a 1:1 ratio, centrifuged at 1,600 g for 10 min, and then the bacteria were allowed to stand on the bench top at room temperature. After the bacteria had incubated together in the centrifuged pellets, the pellets were resuspended, and then plated on LB + 6 μg/mL ceftazidime and 20 μg/mL chloramphenicol, and the plates were inspected on the UV transilluminator box for double-resistant, green-fluorescing colonies. In pilot experiments, we tested mixing the two bacterial strains for 3, 4, and 6 h, but we never observed any transconjugant colonies at these durations of bacterial contact. Finally, we therefore allowed the centrifuged bacterial mixtures to stand on the bench top at room temperature for 20 h, and with this longer duration we did observe putative transconjugant colonies, meaning double-resistant, green-fluorescing colonies. Putative transconjugant colonies were picked and re-streaked on LB with ceftazidime + chloramphenicol. Biochemical tests were next performed to determine if the putative transconjugant colonies were *E. coli* that had gained resistance to ceftazidime, or *E. cloacae* that had developed chloramphenicol resistance and also acquired the plasmid expressing GFP. The biochemical tests done to distinguish *Enterobacter* from *E. coli* included indole production, acid production by the Methyl Red test, Voges-Proskauer test for acetoin production, and streaking on eosin methylene blue (EMB) agar. In all cases the putative transconjugants were *E. coli* that had become resistant to ceftazidime.

### Testing for beta-lactamase-encoding genes in *enterobacter* and in *E. coli* transconjugants

We extracted total DNA from *Enterobacter* E_clo_Niagara and from the putative *E. coli* transconjugants that had acquired ß-lactam resistance using the AllPrep Bacterial DNA Kit from Qiagen (Hilden, Germany and Waltham, MA) according to the manufacturer's instructions. Purified DNA was sent to ID Genomics, Seattle, WA, for PCR detection of the extended spectrum ß-lactamase genes. PCR assays using broad-range primers initially identified the ß-lactamase as a member of CTX-M family, and then specific primers indicated the ß-lactamase was a CTX-M27. This “head start” from ID Genomics allowed us to then conduct our own PCR assays.

### PCR detection of CTX-M27 in *E. cloacae* and in putative *E. coli* transconjugants/transformants

Plasmid DNA was prepared from the donor strain, E_clo_Niagara, recipient strain EC43, and from the putative transconjugant *E. coli* strains that emerged from the inter-species gene transfer experiments, using Qiagen Midi-Plasmid preparation kits (Waltham, MA). Primer pairs directed against the gene *bla*_CTX−M27_, encoding the extended spectrum beta-lactamase CTX-M27, were used for PCR and are shown in Table 2A. PCR was conducted on a Bio-Rad CFX96 real-time PCR machine using Bio-Rad iTaq Universal SYBR Green Master Mix in a final volume of 20 μL; Primer concentration was 0.5 μM, and 5 μL of the plasmid DNA prepared from each strain was used as the template for 35–39 PCR cycles using a 3-Step amplification protocol.

**Table 2:** Oligonucleotides used.

**(A)** PCR primers used to amplify plasmid DNA for CTX-M27 beta lactamase.		
**Primers**	**Amplicon size, b.p.Comment**
Forward CTGGAGAAAAGCAGCGGAG Reverse TGCTTTTGCGTTTCACTCTG	158	From (Szczepanowski et al., 2009), Supplemental Data
Forward GCGACAATACCGCCATGAAC Reverse CGTATTGCCTTTGAGCCACG	257	Designed using Primer BLAST tool based on Accession No. AY156923, from Bonnet (2004) (https://www.ncbi.nlm.nih.gov/)
**(B)** Fluorescently labeled oligonucleotide used for electrophoretic mobility shift assay.		
This 38-mer oligonucleotide was labeled at the 5′ end with 6- FAM (fluorescein)		
GGCCACGCGTCGACTAGTACTTTTTTTTTTTTTTTTTV		

### Electrophoretic mobility shift (EMSA; “Gel-Shift”) assay for recA binding to ssDNA

Purified RecA protein from NEB was used for EMSA along with 10X Assay buffer (Tris acetate, pH 7.8, with magnesium) supplied by the manufacturer. To test for binding to ssDNA, a fluorescently labeled oligonucleotide was purchased from Integrated DNA Technologies, Inc., Coralville, IA. This 38-mer oligonucleotide was labeled at the 5′ end with 6-FAM (fluorescein) and the sequence is shown in Table 2B.

To test for RecA-DNA binding, we first did pilot experiments to determine how much fluorescent oligonucleotide was needed to produce a visible fluorescent band on agarose gels. We found that using 5 μM fluorescent oligonucleotide gave a visible band when 15 μL were loaded on an agarose gel. We next did pilot experiments to determine what molar ratio of RecA protein to oligonucleotide was needed to observe any gel shift.

The binding assay was carried out in duplicate or triplicate in 15 μL final volume in conical-bottomed 96-well plates kept on ice during reagent addition. The fluorescent oligonucleotide in assay buffer was added first, followed by zinc or other metals added to final concentrations of 0.1–1 μM. In addition, ATP or ATP-γ-S was added as noted to achieve a final concentration of 0.3 mM. Last, RecA protein was added from a concentrated stock to achieve the appropriate ratio relative to ssDNA. This ratio was varied to obtain RecA: oligonucleotide ratios of 2:1 to 5:1, as indicated in the Figure Legends. After addition of RecA, the plate was moved to the 37° incubator where it was sandwiched between pre-warmed metal blocks to speed heat transfer to the samples, and binding was allowed to proceed for 15 min. After the incubation, 2 μL of 10 X “Blue Juice” loading dye was added to the samples, and then samples were loaded into the wells of an agarose gel (1.5% agarose in 1 X TAE buffer, with SYBR-Safe DNA stain added to be able to visualize the DNA ladder).

Electrophoresis was carried out at 135 V for 35 min. After electrophoresis, the gel was visualized on the Gel-Doc E-Z fluorescent imager (Bio-Rad) using the Blue illuminator plate and the settings for SYBR Green, which also work for fluorescein. In some experiments, zinc or other metals were added to the agarose gel at the same concentration as that added to the binding assay. In this case, we used plastic rulers cut to the correct length in order to have some samples running in gel lanes with metal incorporated into the gel itself (Supplemental Figure 1). For quantitation, the image files from the Gel-Doc EZ were “inverted” to produce dark bands on a light background, then quantitated using Un-Scan-It Gel software for the MacIntosh computer (Silk Scientific, Orem, UT).

### Measurement of extracellular DNA

DNA released into culture supernatants by SOS-inducing treatments was measured in a UV spectrophotometer by A_260_/A_280_ ratio.

### Statistical analysis

Data shown are means ± SD. Tests of significance were by analysis of variance (ANOVA), using Dunnett's test for multiple comparisons. Graphs were produced using GraphPad Prism (San Diego, CA).

## Results

### Testing for induction of hypermutation in *enterobacter cloacae* strain E_clo_Niagara

**Figure 1:**
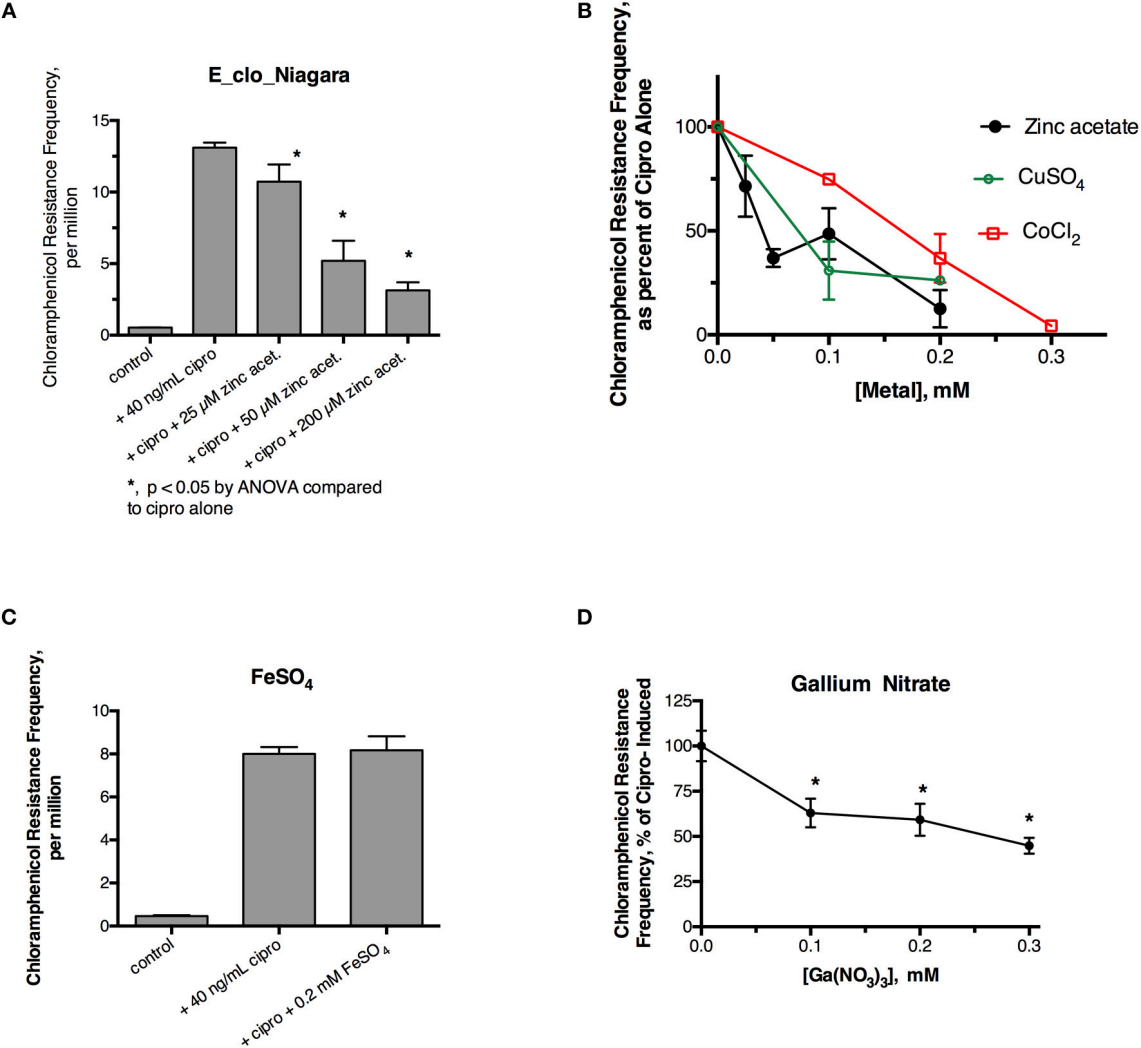
Effect of zinc and other metals on ciprofloxacin-induced hypermutation in E_clo_Niagara. **(A)** Effect of ciprofloxacin and zinc on the chloramphenicol resistance frequency in E_clo_Niagara. **(B)** Comparison of zinc with CuSO_4_ and CoCl_2_ on ciprofloxacin -induced mutation frequency. To allow comparison of separate experiments, the results were expressed as a percent of the ciprofloxacin-induced mutation frequency for each experiment, then averaged. Each line on the graph shows the mean ± SD of at least two separate experiments. **(C)** Lack of effect of FeSO_4_ on ciprofloxacin induced hypermutation. **(D)** Effect of gallium (III) nitrate on ciprofloxacin-induced hypermutation in E_clo_Niagara. ^*^Significant compared to cipro alone by ANOVA; graph shown is the Mean ± SD of 3 separate experiments, expressed as % of cipro-induced.

Initial experiments were performed to see if ciprofloxacin could induce hypermutation to the unrelated antibiotic, chloramphenicol, in E_clo_Niagara. For these experiments we used a chloramphenicol concentration five times the starting MIC for this strain. Figure 1A shows that a sublethal concentration of ciprofloxacin did greatly increase the chloramphenicol resistance frequency in this strain, and this increase was reversed by zinc acetate in a dose-dependent fashion. As previously observed, however, 200 μM zinc acetate was needed to observe strong inhibition of the mutator response (Figure 1A). In addition to zinc, CuSO_4_, and CoCl_2_ also were able to inhibit the hypermutation response (Figure 1B), but they were less potent than zinc, as we previously observed with *E. coli* and *Klebsiella* (Bunnell et al., 2017). In contrast to zinc, iron (as FeSO_4_), and manganese did not inhibit ciprofloxacin-induced hypermutation (Figure 1C; data not shown for MnCl_2_). Interestingly, gallium nitrate also had a mild inhibitory effect on ciprofloxacin-induced hypermutation, even though gallium is a trivalent rather than divalent element.

We next tested whether metal ionophores, such as A23187, or zinc pyrithione, could improve the potency of zinc against the hypermutation response. Our results with A23187 plus zinc were unexpectedly negative, because A23187 alone, in the absence of any metal, consistently increased ciprofloxacin-induced mutation to chloramphenicol resistance (Supplemental Figure 2A, middle bar). Compared to zinc acetate alone, the addition of 100 μM A23187 unexpectedly reduced the potency of zinc (dose-response curve shifted to the right, Supplemental Figure 2B). These results may be consistent with A23187 acting more as a zinc chelator than as a zinc ionophore in E_clo_Niagara. Indeed, most of the literature on A23187 in bacterial systems has been with Gram-positive rather than Gram-negative bacteria, and it may be that A23187 is unable to conduct zinc across the double membranes of enteric Gram-negative bacteria.

**Figure 2:**
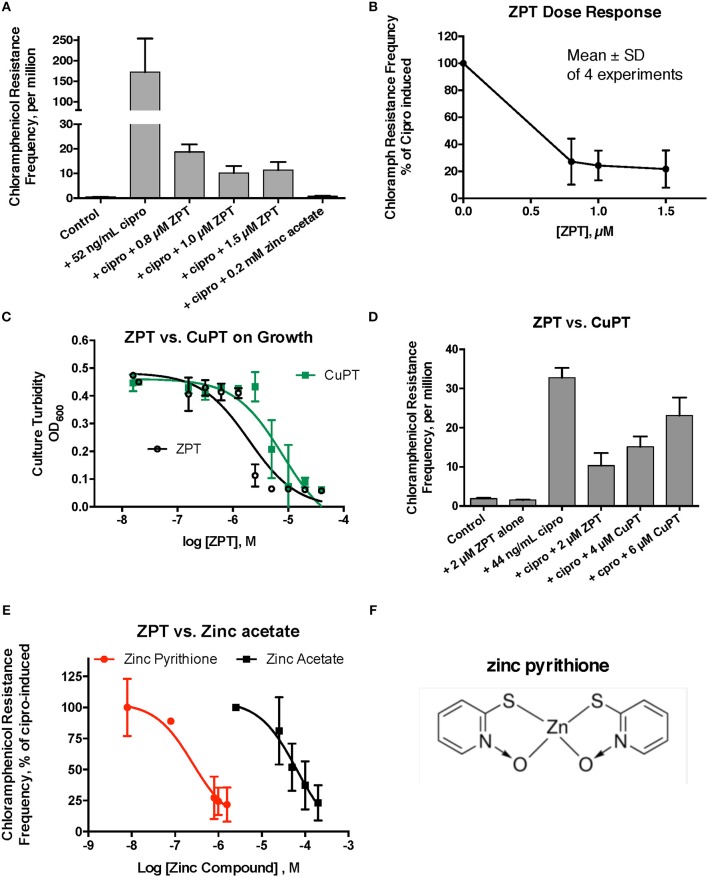
Effect of zinc pyrithione (ZPT) on ciprofloxacin–induced mutation response in E_clo_Niagara. ZPT is also known as 1-hydroxypyridine-2-thione zinc salt. **(A)** Dose-response relationship of ZPT on ciprofloxacin-induced mutation to chloramphenicol resistance, showing a single representative experiment. **(B)** Dose-response relationship of the ZPT on chloramphenicol resistance frequency, showing combined results from 4 separate experiments. ZPT at concentrations greater than or equal to 0.8 μM produced statistically significant reductions in chloramphenicol resistance frequency in **(A,B)** (< 0.05). **(C)** Comparison of ZPT and copper pyrithione (CuPT) on growth of E_clo_Niagara. **(D)** Comparison of ZPT and CuPT on inhibition of chloramphenicol resistance. Although 2 μM CuPT produced a partial inhibition of hypermutation, higher concentrations of CuPT showed a paradoxical increase back toward the ciprofloxacin-induced level; this was due to increased killing of bacteria at 4–6 μM, resulting in a drop in the denominator value (total counts). **(E)** Dose response relationship of zinc compounds in inhibition of mutation to chloramphenicol resistance, expressed on a log scale. ZPT curve is the mean ± SD of 4 experiments, while the zinc acetate curve is the mean ± SD of 7 separate experiments. **(F)** Chemical structure of zinc pyrithione.

Despite the unexpected results with A23187 + zinc, zinc pyrithione (ZPT) showed much more promise as an inhibitor of SOS-induced hypermutation. ZPT inhibited ciprofloxacin-induced hypermutation at concentrations near 1 μM (Figures 2A,B). While copper pyrithione (CuPT) is often viewed as more toxic to living cells than ZPT (Bao et al., 2014), we found that ZPT was ~4-fold more potent than CuPT in its ability to inhibit bacterial growth (Figure 2C). Similarly, ZPT was better than CuPT in blocking the mutator response (Figure 2D). Direct comparison of zinc acetate to ZPT showed that ZPT was ~100 fold (2 logs) more potent than zinc acetate in inhibition of SOS-induced hypermutation (Figures 2E,F). The high potency of ZPT against the SOS response might allow the effects of zinc to be exploited in situations where concentrations of zinc acetate as high as 0.2 mM cannot be achieved.

### Testing for horizontal, inter-species gene transfer in enteric bacteria

Having shown that the SOS response could be induced in E_clo_Niagara, we next tested whether SOS induction could trigger transfer of antibiotic resistance elements between species, as shown by Beaber et al. (2003). In contrast to the study by Beaber et al., who worked with *V. cholerae*, an organism that is naturally competent for uptake of extracellular DNA, *E. coli* and related enterics are not naturally competent, and although they can take up DNA from the environment, such DNA is often degraded and used as food rather than incorporated into their genomes (Finkel and Kolter, 2001; Chen and Dubnau, 2004; Palchevskiy and Finkel, 2006). Therefore, it was unclear whether the findings of Beaber et al. on SOS and horizontal gene transfer would apply to a different microbial system.

**Figure 3:**
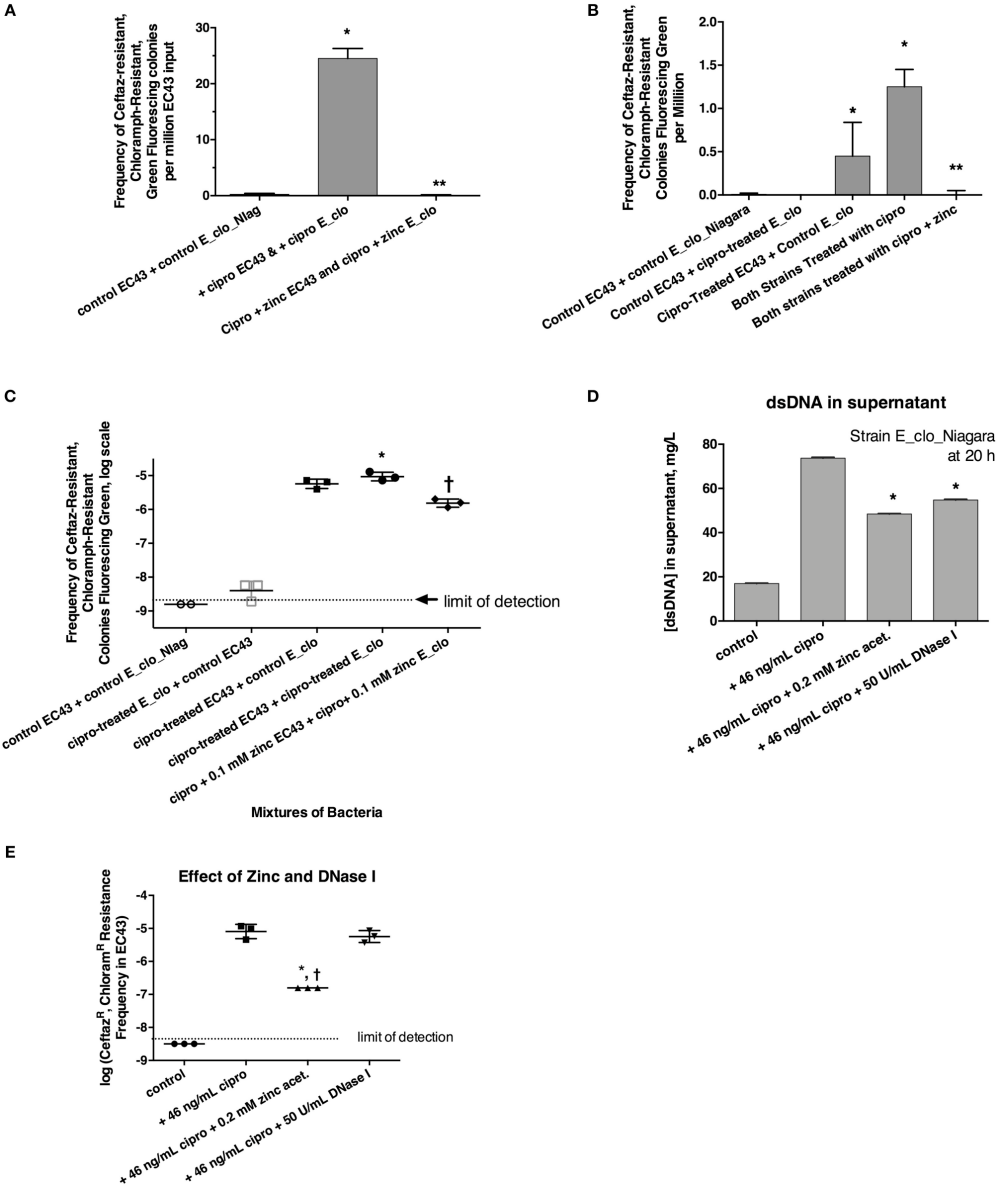
Role of the SOS response in horizontal transfer of beta-lactam resistance from E_clo_Niagara to susceptible *E. coli* strain EC43. **(A)** Induction and inhibition of inter-species gene transfer. E_clo_Niagara and *E. coli* EC43 were both treated separately with ciprofloxacin to induce the SOS response, then mixed and allowed to co-incubate for 20 h. The mixtures were plated on LB + ceftazidime + chloramphenicol, and examined under UV light for green fluorescing colonies resistant to both antibiotics. Since the colonies fitting the above description all tested as being *E. coli*, not Enterobacter, the frequency of emergence of such colonies was calculated relative to the number of EC43 bacteria placed into the original mixture. Zinc acetate concentration was 0.2 mM; ^*^significantly increased compared to control, ^**^significantly decreased compared to the conditions receiving ciprofloxacin. **(B)** Effect of ciprofloxacin applied to E_clo_Niagara alone, *E. coli* EC43 alone, or both strains, on the horizontal gene transfer. The concentration of zinc used in **(B)**, far-right column, was 0.2 mM; ^*^significantly increased compared to control, ^**^significantly decreased compared to the conditions receiving ciprofloxacin. **(C)** Effect of ciprofloxacin and zinc on horizontal gene transfer, with the transconjugant frequency expressed on a log scale. 0.1 mM zinc was used in panel C; ^*^significantly increased compared to control, †significantly decreased compared to the conditions receiving ciprofloxacin. **(D)** Effect of zinc and DNase I on release of double-stranded DNA into culture supernatant; ^*^significantly decreased compared to the plus cipro condition. **(E)** Effect of zinc and DNase I on frequency of emergence of putative transconjugants (green fluorescing colonies resistant to both antibiotics), showing that zinc, but not DNase, blocked the transfer of beta-lactam resistance to the *E. coli* strain. ^*^significantly decreased compared to the condition receiving ciprofloxacin alone. †There were no Ceftaz^R^, Chloram^R^ *E. coli* colonies in the zinc-treated group, so the data points shown are calculated as if 1 colony had appeared.

We tested whether the ß-lactamase of *Enterobacter* E_clo_Niagara could be transferred to a susceptible STEC *E. coli* strain, EC43. EC43 also possessed its own plasmid, encoding the green fluorescence protein (GFP) and resistance to chloramphenicol. As described in section Materials and Methods, we induced the SOS response in each bacterial strain, then mixed together the 2 strains of bacteria at a 1:1 ratio, and, after allowing the mixtures to incubate for increasing periods of time, then plated aliquots on LB agar + chloramphenicol + ceftazidime. Plates were examined on the UV transilluminator box for green-fluorescing colonies resistant to both antibiotics. In initial experiments where the E_clo_Niagara and EC43 were allowed to co-mingle for 3, 4, or 6 h before plating on antibiotic selective agar, no green fluorescing colonies were observed on the double-antibiotic plates. When the 2 species of bacteria were allowed to co-incubate for 20 h, however, green colonies resistant to both antibiotics were observed in the condition in which the SOS-response was induced with ciprofloxacin (Figure 3 and Supplemental Figure 3A). Addition of 0.2 mM zinc acetate blocked the appearance of the putative transconjugants when zinc was added to both bacteria along with ciprofloxacin (Figure 3A). Identification of the double-resistant, green colonies showed them to be *E. coli* that had acquired resistance to ceftazidime, rather than E_clo_Niagara that had acquired the plasmid encoding GFP and the chloramphenicol resistance marker. Control experiments with EC43 alone, not mixed with E_clo_Niagara, failed to show ciprofloxacin–induced acquisition of ceftazidime resistance (zero colonies observed in 10 separate experiments, all done in triplicate).

We next induced the SOS response in EC43 and E_clo_Niagara separately to try to determine if SOS induction was more important in the donor strain or the recipient strain. When only the donor strain, E_clo_Niagara, was treated with ciprofloxacin, putative transconjugants remained rare (Figures 3B,C, respectively). When only *E. coli* EC43 was subjected to SOS induction with ciprofloxacin, putative transconjugants were regularly observed in substantial numbers (Figures 3B,C). The greatest frequency of transconjugants was observed, however, when both the donor and the recipient strain were treated with ciprofloxacin (Figures 3B,C). Zinc acetate consistently blocked the SOS-induced transfer of ß-lactam resistance phenotype. When the zinc concentration was reduced to 0.1 mM, however, the reversal of the SOS response was only partial (Figure 3C, right-hand set of points).

In the course of these experiments, we noted that induction of the SOS with ciprofloxacin, followed by a 20 h incubation, was accompanied by a release of DNA into the supernatant (Figure 3D; similar results were obtained with strain EC43 as well; data not shown). During the 20 h incubation, bacterial counts of both strains increased ~3-fold compared to the counts at the 4 h collection point, for both the antibiotic-treated and untreated conditions. Therefore, the DNA release observed is not due to massive die-off of bacteria during the 20 h incubation. Addition of zinc acetate and DNase I both significantly reduced the amount of DNA detectable in bacterial supernatants (Figure 3D). Therefore, we tested whether addition of exogenous DNase I would block the horizontal transfer of ß-lactam resistance to EC43. As shown in Figure 3E, DNase I had no effect on the frequency of appearance of the double-resistant transconjugant colonies in this assay, while zinc again showed its strong inhibitory effect. This also hinted that, although release of DNA into the supernatant occurred during our 20 h incubation, transfer of relevant DNA encoding antibiotic resistance likely does not occur by bulk release of DNA into the culture medium, but instead may require direct cell-to-cell contact.

The experiments of Figure 3 showed that the beta-lactam resistant phenotype from E_clo_Niagara, could be transferred to a susceptible *E. coli* strain when the SOS response was induced. We next tested whether we could detect the gene encoding the resistance had indeed been transferred to the recipient strain. The extended spectrum beta-lactamase (ESBL) gene family is large, but the ID Genomics company helped us narrow our focus to *bla*_CTX−M27_. Using this knowledge, we constructed two sets of primers directed against CTX-M27 (section Materials and Methods and Table 2). PCR with both sets of primers revealed that DNA encoding CTX-M27 was present in the donor strain E_clo_Niagara and in the *E. coli* transconjugants, but not present in parental *E. coli* strain EC43. Quantitative PCR analysis from a typical experiment showed that the number of cycles to threshold, C_t_, for amplification from the plasmid preparations from the various strains were: E_clo_Niagara: 28.0 ± 0.7; EC43: NA (no amplification); Transconjugant 1: 32.2 ± 1.0; Transconjugant 2, 27.3 ± 0.5; and Transconjugant 3, 35.8 ± 0.3, using the first set of primers shown in Table 2. Similar results were observed using the second set of primers as well.

### RecA-ssDNA-Zinc interactions

**Figure 4:**
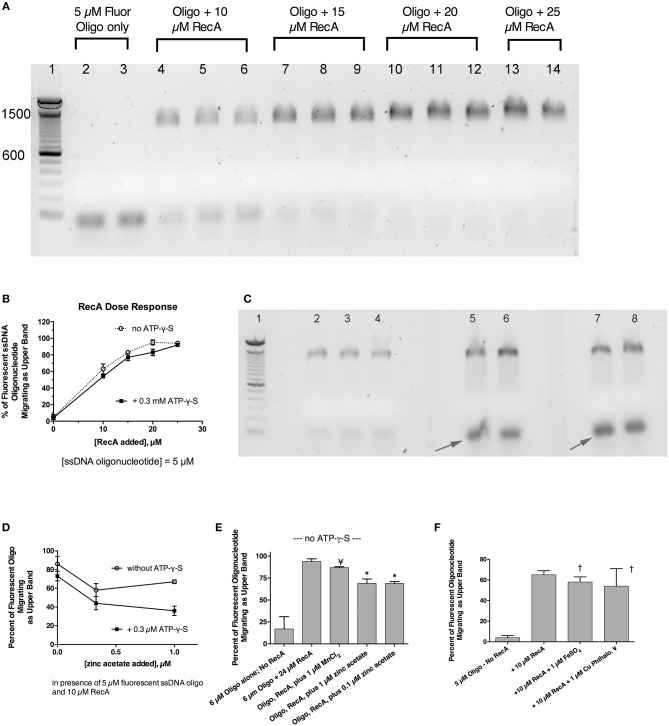
Effect of zinc and other metals on the binding of RecA protein to a fluorescently labeled single-stranded DNA probe. Purified RecA protein and a 38-nucleotide single-stranded DNA probe labeled with fluorescein were used in an electrophoretic mobility shift assay (EMSA) in 1.5% agarose gels. **(A)** Effect of increasing amounts of RecA on the migration of the fluorescently labeled ssDNA. Bands shown in **(A,C)** are the fluorescent ssDNA, but the digital image of the gel was “inverted” to produce dark bands on a white background. Lane 1, 100 bp dsDNA molecular weight ladder, with the bands 600 and 1,500 pairs indicated. Lanes 2 and 3, 5 μM fluorescent oligonucleotide alone, with no RecA. Lanes 4–6, ssDNA + 10 μM RecA; Lanes 7–9; ssDNA + 15 μM RecA. Lanes 10–12, plus 20 μM RecA; Lanes 13–14, with 25 μM RecA. **(B)** Quantitative gel scan of the gel shown in **(A)**, as well as an additional gel done in the presence of ATP-γ-S (latter gel not shown), showing that as the RecA protein is increased, the percent of the ssDNA migrating as the upper band increases. **(C)** Effect of zinc on the gel shift induced by RecA protein, in the presence of 0.3 mM ATP-γ-S. Lane 1, 100 bp DNA MW ladder; Lanes 2–8 all received 5 μM ssDNA probe + 10 μM RecA (2:1 ratio of RecA to ssDNA). Lanes 5 & 6, + 0.3 μM zinc acetate; Lanes 7 & 8, + 1 μM zinc acetate. Zinc was also cast into the appropriate lanes of the agarose gel as described in section Materials and Methods and Supplemental Figure 3. **(D)**, quantitative gel scans of the gel shown in **(C)**, plus another gel done in the absence of ATP-γ-S, are graphed as a function of the concentration of added zinc. **(E)** Comparison of the effect of zinc on the gel shift assay, in contrast with the lack of effect of MnCl_2_. With RecA added to achieve a 4:1 ratio relative to ssDNA, the inhibitory effect of 1 μM zinc was still evident, while 1 μM MnCl_2_ failed to inhibit the RecA-induced upward shift in the migration of the ssDNA probe; ¥, not significant compared to Oligo + RecA, by ANOVA; ^*^significant compared to Oligo + RecA; *p* < 0.01 by ANOVA. **(F)** Lack of effect of 1 μM FeSO_4_ and 1 μM copper phthalocyanine tetrasulfonate (¥), reported to be a RecA inhibitor, on the gel shift induced by RecA. ^†^Not significant compared to RecA alone by ANOVA.

Previously, we showed that zinc blocked the SOS-induced cleavage of the LexA repressor in live *E. coli* bacterial cells, and zinc also blocked RecA-mediated LexA cleavage in a cell-free assay using purified bacterial proteins plus cofactors (Bunnell et al., 2017). That previous work strongly suggested that zinc acted on RecA, as previously suggested by other authors (Lee and Singleton, 2004), but left open a narrow possibility that zinc might act on LexA, or on the protein-protein interface where RecA binds to LexA, possibly interfering with LexA's own proteolytic cleavage. To determine if zinc had effects on RecA alone, in the absence of LexA, we performed EMSAs using fluorescein-tagged ssDNA and purified RecA to see if zinc influenced the ability of RecA to bind to ssDNA (Svingen et al., 2001). Figure 4A shows the effect of adding increasing amounts of RecA protein in the presence of a fixed, 5 μM concentration of fluorescent 38-mer ssDNA oligonucleotide. A marked upward shift in mobility was noted in the presence of 10 μM RecA (lanes 4–6, 2:1 molar ratio of RecA to ssDNA), and the proportion of ssDNA migrating in this slower, upper band increased as the ratio of RecA to ssDNA was increased (Figure 4A, lanes 7–14). Figure 4A shows the results obtained in the absence of ATP-γ-S, but experiments in the presence of 0.3 mM ATP-γ-S were also performed, and the densitometry scans of the fluorescence images, with and without ATP-γ-S, are shown in Figure 4B. Addition of zinc markedly reduced the proportion of ssDNA migrating as the upper band, and increased the amount of ssDNA in the original, unbound position (black arrows). Gels scans of Figure 4C and a parallel experiment done in the absence of ATP-γ-S are shown in Figure 4D. As shown in Figure 4D, the inhibitory effects of zinc on RecA binding were enhanced in the presence of ATP-γ-S, consistent with the known role of ATP and ATP-γ-S in supporting the dissociation of bound RecA monomers from the ssDNA nucleofilament (Wigle et al., 2006). The inhibitory effects of zinc on RecA binding persisted even when higher concentrations of RecA were used in the assay (Figure 4E; 4:1 ratio of RecA to ssDNA). In contrast to the inhibitory effects of zinc, manganese did not inhibit ssDNA binding to RecA (Figure 4E), and neither did 1 μM FeSO_4_ (Figure 4F). Surprisingly, the newly discovered RecA inhibitor, copper phthalocyanine tetrasulfonate, also failed to inhibit RecA-ssDNA binding in this assay (Figure 4F). The 1 μM concentration used in Figure 4F, however, was lower than the 10–15 μM concentrations used in the report by Alam et al. however (Alam et al., 2016). Nevertheless, Figure 4 shows that RecA binding to ssDNA can be readily detected using a fluorescently labeled oligonucleotide in agarose gels, and that zinc inhibits RecA binding to ssDNA at low, 1 μM concentrations, while iron, manganese, and a phthalocyanine tetrasulfonate compound did not. In addition, although zinc acts to block RecA-induced LexA cleavage (Bunnell et al., 2017), the presence of LexA is not necessary to observe the effects of zinc *in vitro*. Our results suggest that zinc blocks LexA cleavage by preventing the formation of the active RecA-ATP-ssDNA nucleofilament.

## Discussion

Although the SOS response has been discussed in the molecular biology literature for over 40 years (Radman, 1975), appreciation of the role of the SOS pathway in generation of antibiotic resistance has been very slow to percolate into the fields of clinical microbiology and infectious diseases. Recently, however, several laboratories have shown that sublethal concentrations of antibiotics can not only induce the SOS response, but also trigger the emergence of resistance to other, unrelated antibiotics (Kohanski et al., 2010; Song et al., 2016). In addition, our laboratory and others have shown that SOS-induced hypermutation, resulting in the emergence of antibiotic resistance, can be triggered by a wider array of compounds than classical inducers such as mitomycin C, ciprofloxacin, and UV light. The SOS response is induced in *E. coli* by oxidant stress and by a wide variety of drugs used in human and veterinary medicine, including some antiviral drugs, cancer chemotherapy agents, antimetabolites, arsenic compounds, the herbicide paraquat, and many other antibiotics, including those used for growth promotion in farm animals (Baharoglu and Mazel, 2011; Bunnell et al., 2017). We began studying zinc because it is an inhibitor of Shiga toxin (Stx) production in STEC, but later learned that zinc was blocking the SOS response itself, not just Stx toxin release. We showed that zinc, but not most other metals tested, blocked SOS-induced hypermutation to antibiotics such as rifampin and trimethoprim, in *E. coli* and *Klebsiella pneumoniae* (Bunnell et al., 2017). As expected from the work of others (Cirz and Romesberg, 2006; Recacha et al., 2017), the hypermutation response, and zinc's inhibitory effects on SOS-induced mutation were dependent on RecA, a key sensor of DNA damage and initiator of the SOS response (Bunnell et al., 2017).

In this study, we first tested whether the inhibitory effects of zinc could be observed in another member of the Enterobacteriaceae family, *E. cloacae*. Zinc blocked ciprofloxacin-induced hypermutation, reducing the emergence of chloramphenicol resistance, while cobalt, copper, and gallium showed similar, but weaker activity (Figure 1). We also showed that a zinc ionophore, zinc pyrithione (ZPT), blocked SOS-induced hypermutation with an inhibitory concentration 50% (IC_50_) ~100-fold lower than zinc salts. This high potency compares favorably with other RecA inhibitors that have been proposed (Lee et al., 2005; Wigle et al., 2009; Alam et al., 2016). Although ZPT can have some toxicity toward mammalian cells (Priestley and Brown, 1980), it has been cleared by the United States Food and Drug Administration (FDA) for use on the skin at concentrations up to 0.25% (7.8 mM) in “leave on” products such as lotions, and 2% (63 mM) in “rinse-off” products such as shampoos (Schwartz, 2016). Older literature on ZPT tended to focus on its antifungal effects (Pierard-Franchimont et al., 2002; Reeder et al., 2011), but more recent reports emphasize the activity of ZPT against bacteria as well (Schwartz, 2016).

Inspired by the work of Beaber et al., we tested whether induction of the SOS response would trigger horizontal gene transfer from a ß-lactam resistant, ESBL-producing *Enterobacter* strain to a sensitive *E. coli* strain. We were able to demonstrate transfer of the ß-lactamase gene to the *E. coli* EC43 strain after a prolonged, 20 h, co-incubation. Although we have referred to the newly-resistant EC43 colonies as “putative transconjugants,” we have not conclusively shown that the mechanism of transfer of the ß-lactamase is by conjugation. Jung tested a panel of 15 *Enterobacter aerogenes* strains for their ability to conjugate with *E. coli* and found only 1 of the 15 strains capable of doing so (Jung, 2014). Indeed, when we tested for transfer of the AmpC chromosomal ß-lactamase from a different *E. cloacae strain* (BAA-1143) to EC43, putative transconjugants were either much rarer, or, in some experiments, not observed at all (data not shown). The rare transconjugants observed with strain BAA-1143 as the donor were also unstable genetically, and gradually lost their ß-lactam resistance with repeated passage.

PCR analysis of the *E. coli* transconjugants that had acquired ceftazidime resistance revealed that the CTX-M27 beta-lactamase gene was present in the plasmid DNA. Therefore, it may be more accurate to describe these resistant *E. coli* strains as transformants (Chen and Dubnau, 2004) rather than as transconjugants. Classically, conjugation proceeds via transfer of ssDNA via a conjugation pilus to the recipient strain, whereas transformation (such as by electroporation) is usually more efficient with double-stranded plasmid DNA. McCollister et al. recently described *in vivo* acquisition of the CTX-M27 plasmid by a *Salmonella enterica* strain in a relapsed infection (McCollister et al., 2016). In that case, the patient's *Salmonella* strain was initially susceptible to the 3^rd^-generation cephalosporins and the patient was treated with ceftriaxone. After relapse, however, the *Salmonella* strain was resistant to ceftriaxone, and comparison of the two strains allowed the investigators to determine that the *Salmonella* had acquired the CTX-M27 beta-lactamase, incorporated into a plasmid. The resistance element in *Salmonella* could be transferred back into a susceptible *E. coli* strain, where it also was located on a plasmid. Although we have not conducted the detailed DNA sequencing done by McCollister et al., we believe that our results may reflect a similar process, but with gene transfer between *E. cloacae* and *E. coli*. Notably, beta-lactam antibiotics such as ceftriaxone do induce the SOS response (Drago et al., 2004; Maiques et al., 2006), and so SOS induction could have played a role in the *in vivo* transfer of the resistance gene in the GI tract of the patient described by McCollister et al. It is also notable that the work of McCollister et al. (2016) and Beaber et al. (2003), and the work in this study all used wild-type enteric pathogens in their studies of DNA transfer between species. Over-reliance on highly passaged laboratory strains of *E. coli* in the past may have prevented earlier recognition of these phenomena.

Although DNA, especially ssDNA, is often degraded upon entry into *E. coli* cells via the competence (Com) gene locus (Finkel and Kolter, 2001; Palchevskiy and Finkel, 2006), it is possible that when the SOS is induced, RecA becomes abundant enough that it is able to catalyze recombination of ssDNA with the recipient cell's DNA before the newly acquired DNA is degraded (Beaber et al., 2003). If so, this would explain why transfer of the ß-lactam resistance was greatest when the SOS response was induced in the recipient as well as the donor strain (Figures 3B,C). While the mechanism of the DNA transfer is not clear, what is clear is that transfer is triggered by the SOS response, and that the transfer is strongly inhibited by zinc, raising the possibility that zinc compounds could be used to block horizontal transfer of antibiotic resistance in situations where this gene transfer would be undesirable.

Other authors have suggested that a good way to exploit RecA inhibitors under development would be to reverse pre-existing antibiotic resistance, i.e., in strains already resistant to antibiotics such as the quinolones (Alam et al., 2016; Recacha et al., 2017). We suggest that a more effective strategy would be to use RecA inhibitors, including zinc compounds, to prevent the emergence of resistance in situations where sensitive microbes are still present, but induction of the SOS response would be expected or inevitable, such as when UV light, ionizing radiation, cytotoxic cancer chemotherapy, or other SOS-inducing drugs are used (Rutala et al., 2010).

Although zinc-containing RecA inhibitors show promise for preventing emergence of antibiotic resistance, a concern is whether zinc resistance could emerge as a bacterial counter-measure. Zinc resistance seems to be uncommon in bacteria, but has been reported (McHugh et al., 1975; Hau et al., 2017). Therefore, if RecA inhibitors are ever adopted for use in human or veterinary medicine, animal agriculture, or food processing, prudence and stewardship in use of these RecA inhibitors may become as necessary as prudence and stewardship in the use of antibiotics themselves.

## Author contributions

MC did the initial hypermutation experiments with *E. cloacae* and began the inter-species gene transfer experiments. MO did all of the experiments with zinc ionophores. JC completed inter-species gene transfer experiments, performed the EMSA's, the PCR experiments, and wrote the manuscript. MS helped design experiments, reviewed the manuscript, and made helpful critiques.

### Conflict of interest statement

The authors declare that the research was conducted in the absence of any commercial or financial relationships that could be construed as a potential conflict of interest.
