# Supplementary material for: Zinc Blockade of SOS Response Inhibits Horizontal Transfer of Antibiotic Resistance Genes in Enteric Bacteria
Source: Front Cell Infect Microbiol. 2018 Nov 21;8:410. doi: 10.3389/fcimb.2018.00410 (PMC6258817; doi:10.3389/fcimb.2018.00410)
Supplement: Supplementary file 1 [file Data_Sheet_1.PDF]

# Supplemental Fig. 1

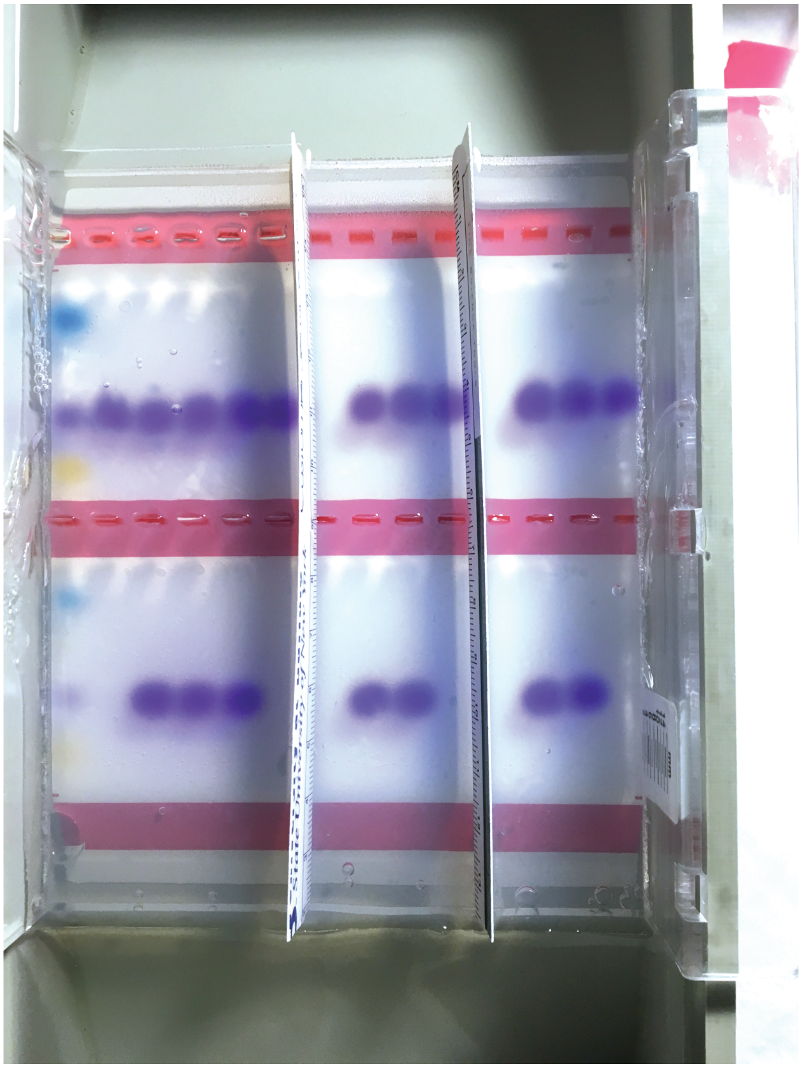

Agarose gel cast in longitutudinal sections, using small plastic rulers as dividers between the sections. Separate batches of 1.5 % agarose were prepared for each section, for example with no zinc in the large section on the left, 0.3 µM zinc acetate incorporated into the agarose in the center section, and 1 µM zinc acetate in the section on the right. Often, an assistant was needed to be able to pour all 3 sections at the same time in the gel casting apparatus.

# Supplemental Figure 2

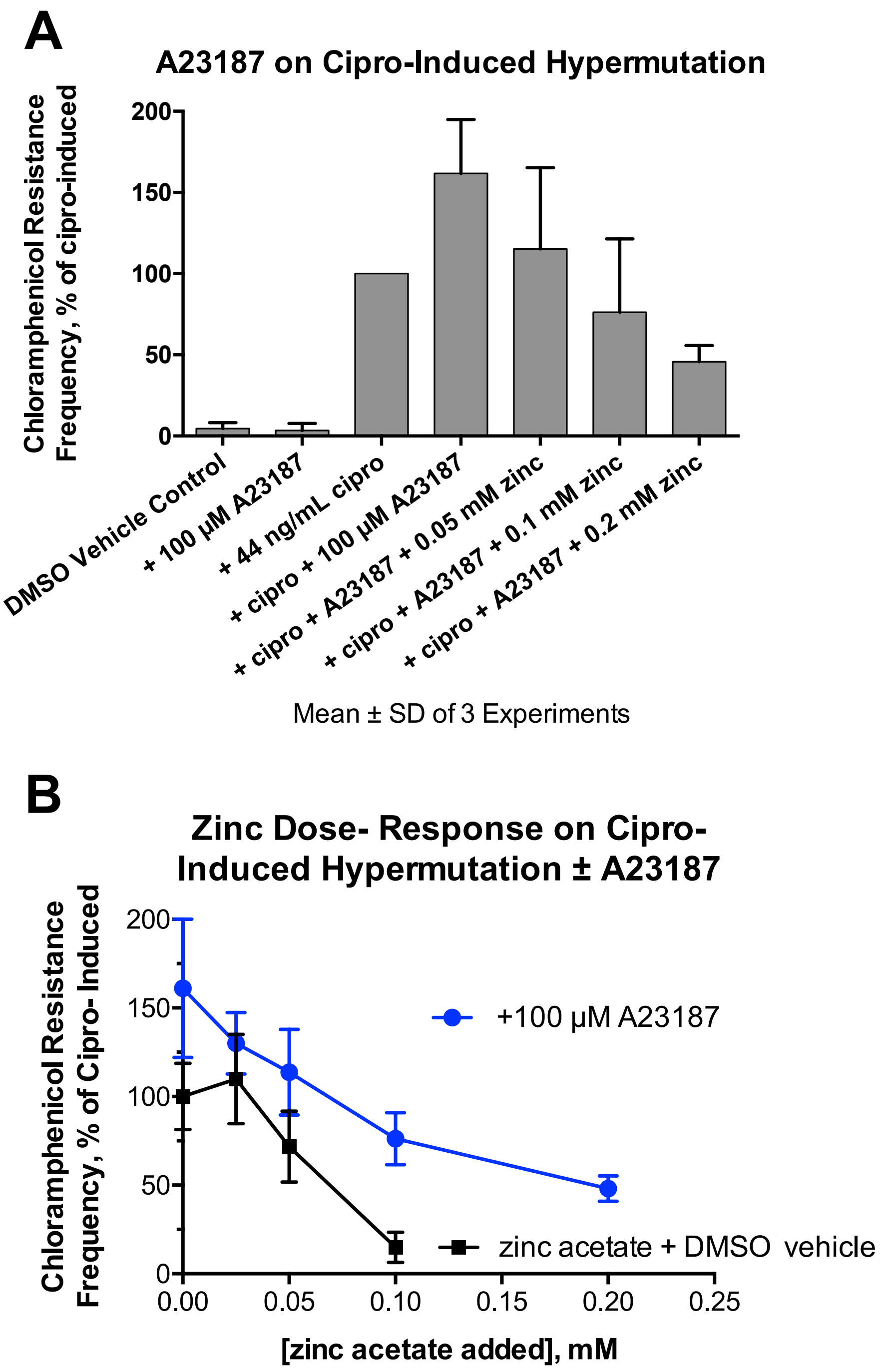

# Supplemental Figure 3

A

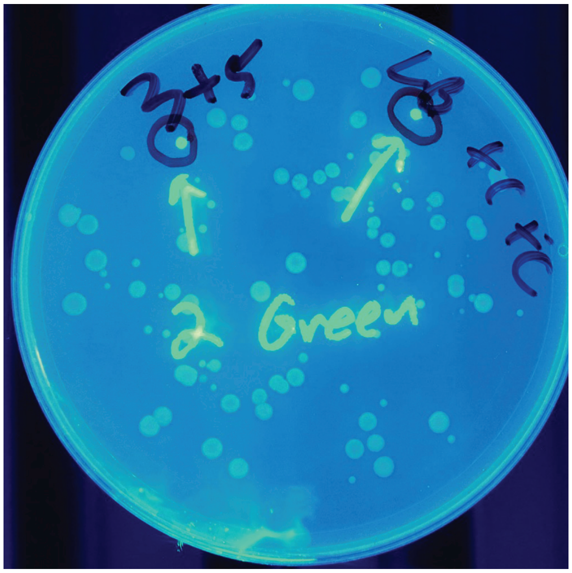


B

Indole Production Using Kovac's Reagent

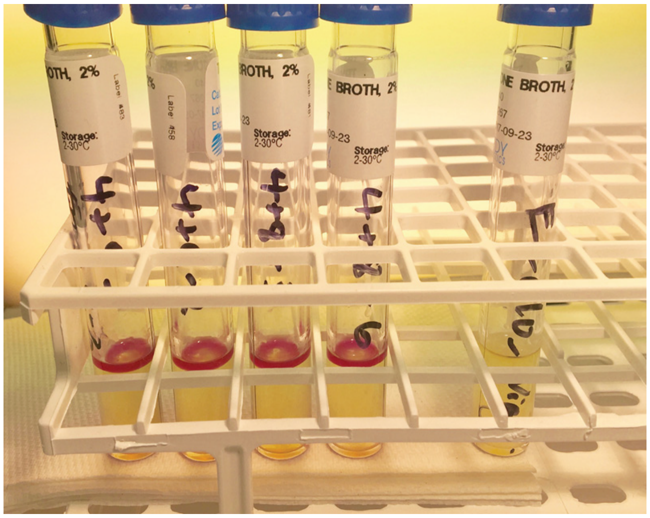

C

Acid Production by Methyl Red Test

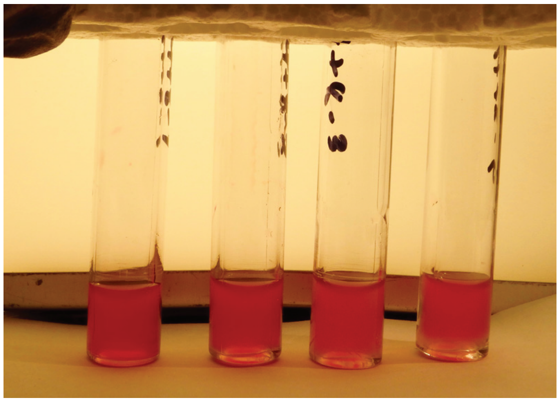

D

EMB Agar Plating for Green Metallic Sheen

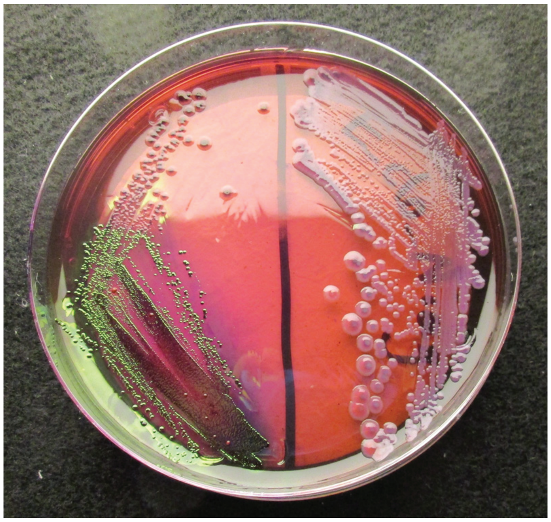

Features Distinguishing *E. coli* from Enterobacter species

| Diagnostic Test | ***E. coli*** | **Enterobacter sp.** |
|---|---|---|
| Indole production from tryptophan | + | - |
| Acid production at 48 h, by Methyl Red test | + | - |
| Voges-Proskauer test for acetoin production | - | + |
| Appearance on EMB agar | **Green metallic sheen** | **No green metallic sheen** |
| Growth at 42 º C | **Grows well** | **Weak or no growth** |
